# Defining a therapeutic range for adalimumab serum concentrations in the management of pediatric noninfectious uveitis, a step towards personalized treatment

**DOI:** 10.1186/s12969-023-00928-2

**Published:** 2023-12-20

**Authors:** Jo L. Dehoorne, Helena Groth, Emma Carlé, Ilse De Schrijver, Celine Sys, Patricia Delbeke, Elke O. Kreps, Thomas Renson, Carolien Bonroy

**Affiliations:** 1 Department of Internal Medicine and Pediatrics, Department of Pediatric Rheumatology, Ghent University Hospital, C. Heymanslaan 10, 9000 Ghent, Belgium; 2 European Reference Network for Rare Immunodeficiency, Autoinflammatory and Autoimmune Diseases at the Ghent University Hospital, Ghent, Belgium; 3 Ghent University, Faculty of Medicine and Health Sciences, Ghent, Belgium; 4 Department of Ophthalmology and Center for Medical Genetics Ghent, Ghent University Hospital, Ghent, Belgium; 5 European Reference Network for Rare Eye Diseases at the Ghent University Hospital, Ghent, Belgium; 6 Department of Ophthalmology, AZ Sint-Jan, Brugge, Belgium; 7 Department of Diagnostic Sciences, Ghent University; Department of Laboratory Medicine, Ghent University Hospital, Ghent, Belgium

**Keywords:** Pediatric noninfectious uveitis, Adalimumab, Therapeutic range, Trough levels, Therapeutic drug monitoring

## Abstract

**Background:**

Adalimumab is currently considered the most efficacious anti-TNFα agent for childhood noninfectious uveitis (NIU). The objective of this study was to define a therapeutic range for adalimumab trough levels in the treatment of childhood NIU.

**Methods:**

A retrospective, observational, pilot study of 36 children with NIU aged < 18 years, treated with adalimumab. Serum adalimumab through levels and adalimumab anti-drug antibodies (ADA) were analysed at least 24 weeks after start adalimumab.

**Results:**

Adalimumab trough levels were significantly higher in complete responders 11.8 μg/mL (range 6.9–33.0) compared to partial or non-responders 9,2 μg/mL (range 0–13.6) (*p* = 0,004). Receiver–operator characteristics analyses with an area under the curve of 0,749 (95% CI, 0,561–0,937) defined 9.6 µg/mL as the lower margin for the therapeutic range. This cut-off corresponds with a sensitivity of 88% and a specificity of 56% (positive predictive value, 85%; negative predictive value, 62.5%). A concentration effect curve defined 13 µg/mL as the upper margin. Approximately one-third (30.5%) of patients had an adalimumab trough concentration exceeding 13 µg/mL. Free ADA were observed in 2 patients (5.5%).

**Conclusions:**

A therapeutic range of adalimumab trough levels of 9.6 to 13 µg/mL, which corresponds with an optimal clinical effect, was identified. Therapeutic drug monitoring may guide the optimisation of treatment efficacy in children with NIU in the treat-to-target era.

**Supplementary Information:**

The online version contains supplementary material available at 10.1186/s12969-023-00928-2.

## Background

Noninfectious uveitis (NIU) in childhood is a chronic potentially sight-threatening condition with an estimated incidence of 4.9 to 30.0 per 100 000 children [1, 2]. Juvenile idiopathic arthritis (JIA) is the most common associated systemic disease (up to 41–47%), whereas 28–51% of cases are idiopathic [3, 4]. Methotrexate is recommended as first choice disease-modifying antirheumatic drug (DMARD) for paediatric uveitis [5, 6]. Though widely used for uveitis, methotrexate fails to control inflammation in nearly 40% of children [7]. The introduction of biological therapies has revolutionized the treatment paradigm of NIU, especially those targeting TNFα. Adalimumab demonstrated its efficacy, particularly in JIA-associated and idiopathic anterior NIU in children [8, 9] and intermediate, posterior and panuveitis in adults [10, 11]. Based on the SYCAMORE trial [9] adalimumab is the only EMA and FDA approved biological agent for paediatric anterior NIU in patients from 2 years of age and currently considered the most efficacious anti-TNFα agent for childhood idiopathic or JIA associated NIU [5]. In analogy with JIA, NIU patients are treated with adalimumab according to a fixed dosing schedule: 20 mg every other week (eow) for patients weighing < 30 kg and 40 mg eow for patients weighing ≥ 30 kg, administered as a subcutaneous injection [9]. With this fixed-dosing regimen, a wide variety in clinical response and adalimumab serum concentrations was observed in children with NIU, with higher serum drug concentrations in responders compared to non-responders [12–14]. This wide inter individual variety,possibly implies that a substantial part of NIU patients are under- or overtreated.

Approximately 30–40% of patients with JIA associated NIU are refractory to both methotrexate and TNF inhibitors, experiencing early treatment failure (primary non-response) or loss of response months after starting treatment (secondary non-response) [15]. Given the limited therapeutic armamentarium of effective biological drugs available and fixed weight-based dosing in childhood NIU, early identification of primary non-response or loss of response is of utmost importance in clinical practice. The therapeutic outcome is closely related to systemic drug levels, which are influenced by several factors such as immunogenicity, concomitant treatment with immunomodulators, genetic factors, anthropometric variables (weight, body surface area) and demographic variables (age, gender, and race) [16, 17]. Drug immunogenicity has been linked to anti-TNFα treatment failure in several inflammatory diseases, including paediatric NIU [12–14]. Development of antidrug antibodies (ADA) to adalimumab, resulting in diminished half-life of the drug and reduced efficacy, have been documented in childhood NIU [12–14]. In this context, emerging evidence is supporting the use of therapeutic drug monitoring (TDM) to optimise biological efficacy, safety and cost-effectiveness of biological agents.

Effective TDM requires the definition of the range of concentrations to which dosing is aimed (i.e. the therapeutic range). Although ranges for serum adalimumab trough levels (TL) have been proposed in the context of several immune mediated inflammatory diseases in adults [18–20], these have not yet been proposed for paediatric rheumatic diseases in general and NIU in particular. Interestingly the new [21] and previous Single Hub and Access point for paediatric Rheumatology in Europe (SHARE) [5] recommendations for the treatment of JIA associated and idiopathic NIU recommend TDM and management changes based on sub-therapeutic drug levels, without actually defining what those levels should be.

Therefore we performed a retrospective cohort study of patients at our institution who had therapeutic drug monitoring (TDM) of their adalimumab therapy. The aim of this pilot study was to report the relationship between serum adalimumab TL and uveitis disease activity in childhood NIU patients treated with a standard dose of adalimumab, providing a concentration effect curve (CEC) and to define a therapeutic range for adalimumab TL corresponding with adequate clinical response. Determination of these values is necessary to compose a therapeutic algorithm for paediatric NIU, in which the dosing schedule could be adjusted according to serum trough levels of adalimumab and ADA.

## Methods

This single-center retrospective pilot study was conducted at the Department of Paediatric Rheumatology and Ophthalmology at the Ghent University Hospital, Belgium, on patients treated with adalimumab for noninfectious uveitis (NIU) from March 2018 to July 2022. In our center all uveitis patients are managed by members of the uveitis team, comprising pediatric ophthalmologist and rheumatologist for extensive infectious and non-infectious evaluation depending on the patient’s history, review of systems, clinical examination and type/location of uveitis. This study was approved by Institutional Ethics Committee of the Ghent University Hospital, Belgium (Approval Number: BC-09577). This was a retrospective medical record review, informed consent was not required.

### Study population and serum samples

Inclusion criteria were: age < 18 years at diagnosis of uveitis, adalimumab treatment for at least 24 weeks and availability of at least one proactive dosage of adalimumab TL (and free ADA, if adalimumab TL were below detection limit) beyond 24 weeks of adalimumab treatment. A minimum treatment duration of 24 weeks was defined to reach steady state, based on pharmacokinetic data of adalimumab in JIA patients [22, 23]. Exception to the 24 weeks limit of treatment duration was applied for those patients developing ADA before this time point.

Subcutaneous adalimumab was administered (eow) at a dose of 20 mg in patients weighing < 30 kg or 40 mg in patients weighing ≥ 30 kg.

Demographic data including age and sex were obtained by medical record review. Ocular examination findings (anatomic location, laterality, complications), diagnosis, time from diagnosis to start adalimumab treatment, weight at performance of TDM, weight corrected adalimumab dose and previous and concomitant systemic treatment were documented. Previous therapy (corticosteroids, methotrexate, cyclosporin, tacrolimus, mycophenolate mofetil) was continued at stable dose with constant method of administration, if tolerated and found necessary at discretion of the treating physician.

In our center, TDM is performed since 2018 in the care of NIU patients treated with adalimumab, with periodic monitoring of serum drug and ADA concentrations in individual patients to allow treatment optimization. TDM is started as of 3 months after initiation of adalimumab and performed approximately 3–4 monthly at regular outpatient clinic visits, scheduled within 24 h prior to administration of the next drug dose. All patients had multiple TDM measurements depending on the length of their follow up. For this particular study, the serum adalimumab TL at steady state, were used for the determination of the therapeutic range. Adalimumab TL were quantified using a bridging ELISA (ApDia Adalimumab kit, reference 710,201). This assay measures concentrations of active drug, that is, drug that is not blocked by ADA (if present) and can still fulfil its function [24]. The assay has a measuring range of 0.5 to 12 µg/mL using the standard pre-dilution of 1:100. Samples with a value above 12 µg/mL at standard dilution, were re-analysed using a pre-dilution of 1:400, according to manufacturer’s instructions. Free ADA were detected using a bridging ELISA (ApDia anti–Adalimumab kit, ref 710,301). This bridging ELISA is a drug sensitive assay implicating that it only measures active unbound ADA [24] and is therefore not capable of measuring ADA in presence of excess of adalimumab. Therefore, free ADA were only determined in patients with unmeasurable adalimumab through levels. The measuring range of the ADA assay is 2,5–125 ng/mL. Observations above the measuring ranges were reported > 125 ng/mL.

### Clinical response

Ophthalmological assessments were performed at baseline and every 3 months after initiation of treatment. If necessary, medical examination was performed more often for non-responders. Ocular evaluations included visual acuity testing (best-corrected visual acuity as measured with Snellen eye chart). Slit-lamp examination to evaluate the anterior segments, anterior chamber cells were graded according to the standardization of uveitis nomenclature (SUN) classification [25]. Intraocular pressure measurements were performed with the Tono-Pen hand-held tonometer (in young children) or the Goldmann applanation tonometer (when possible). Indirect ophthalmoscopy was performed to evaluate the vitreous and posterior segments. Vitreous haze was graded as mild or severe (1–2 + or 3–4 + , respectively). Optical coherence tomography (OCT) was used in all patients to determine the presence of cystoid macular oedema (CME) and/or optic disc swelling. When indicated fluorescein angiography was performed to determine the presence or absence of vasculitis or any abnormal retinal angiographic leakage. For patients with bilateral uveitis, treatment decisions and clinical response reporting were based on the most inflamed eye. At the time of TDM analysis, patients were categorized into complete responders (CR), partial responders (PR) or non**-**responders (NR), based on clinical examination and multimodal imaging, as described by Cordero-Coma al [14]. CR were defined by the presence of grade 0 cells in both anterior and posterior segments and by the absence of any other sign of intraocular inflammation on OCT or angiography. PR were defined by a two-step decrease in anterior chamber cells or vitreous haze, decrease of CME or vasculitis without any finding consistent with the criteria of complete response. NR were defined by a persistent intraocular inflammation without any finding consistent with the criteria of partial response.

### Statistical analysis

Statistical data analysis was performed using SPSS Statistics 22 (IBM Corp). Continuous variables were presented as either mean ± standard deviation or median with interquartile range (IQR), frequency and percentages for categorical variables. Shapiro–Wilk normality tests were performed on continuous variables. For comparison of medians between groups, Mann–Whitney testing was applied. For comparison of proportions, Chi-squared testing was used. Two-sided *p*-values < 0.05 were considered significant.

The adequate lower margin of the therapeutic range for adalimumab TL was estimated by performing a receiver-operator characteristics (ROC) analysis on the adalimumab TL to classify CR versus non CR [26]. The cut-off matching maximum Youden’s index (numerical summary of sensitivity and specificity) was selected as optimal lower cut-off value to differentiate between the group of CR and non-CR.

A concentration effect curve (CEC) was established to identify the upper margin of adequate adalimumab TL corresponding with maximal clinical efficacy. To establish a CEC, all 36 patients were sorted from low to high adalimumab TL, with correlating cumulative % of patients with CR at the specified TL or below (normalized for the maximum % of CR in total patient population).

## Results

### Patient characteristics

 Forty-five adalimumab treated NIU patients were identified in the patient database of the Ghent University Hospital. Thirty-six patients fulfilled the eligibility criteria. Fourteen (39%) male, with a median age of 6.5 years (4.4–10.9) at diagnosis and 10.5 years (6,0–12,0) at the start of adalimumab treatment. The patient characteristics are presented in Table 1.

**Table 1:** Patient Demographics and Characteristics

**Patient Demographics and Characteristics**	Total of patients *N* = 36
Gender (male), n (%)	14 (39)
Age at diagnosis, median, (IQR), years	6.5 (4.4–10.9)
Age at start adalimumab, median (IQR), years	10.5 (6.0–12.0)
Age at adalimumab through level, median (IQR), years	11.3 (7.1–13.0)
Time from diagnosis to adalimumab initiation, median (IQR), months	11.5 (4–33.5)
Time from adalimumab initiation to TL, median (IQR), weeks	37.5 (21–268)
Disease duration at TL, median (IQR), months	25.2 (12–56)
Weight at TL, median (IQR), kg	35 (21–52)
Weight corrected dose at TL, median (IQR), mg/kg body weight	0.85 (0.7–1.1)
**Anatomical location of uveitis n (%)**	
Anterior uveitis	24 (67)
Intermediate uveitis	6 (17)
Posterior uveitis	2 (6)
Panuveitis	4 (11)
Anterior chamber cell count — no. (%)*	
0.5 +	9 (25)
1 +	14 (39)
2 +	12 (33)
3 +	1 (3)
**Laterality, n (%)**	
Unilateral	11 (31)
Bilateral	25 (69)
**Etiological diagnosis n (%)**	
Juvenile idiopathic arthritis	19 (53)
Sarcoidosis	2 (6)
Idiopathic	15 (42)
**Treatment**	
Previous Immunomodulators n (%)	
Oral corticosteroids	13 (36)
Methotrexate	31 (86)
Cyclosporin A	4 (11)
Mycophenolate mofetil	1 (3)
Tacrolimus	1 (3)
Etanercept	1 (3)
Concomitant Immunomodulators n (%)	
Methotrexate	21 (58)
Tacrolimus	1 (3)
**Uveitis complications n (%)**	
Ocular hypertension/glaucoma	3 (8)
Cataract	9 (25)
Band keratopathy	4 (11)
Cystoid Macular edema	11 (31)
Macular epiretinal membrane	3 (8)
Optic disc swelling	5 (14)
Posterior synechia	11 (31)
**Development of ADA, n (%)**	2 (6)

*IQR* Inter Quartile Range, *TL* Through level, *ADA* Antidrug antibodies. In patients with bilateral uveitis, *the eye with higher grade of uveitis was chosen

All patients had active NIU despite treatment with glucocorticoid (systemic or topical) and, all but two patients received at least one other immunosuppressive agent. At the time of TDM, 21 (58%) patients were concomitantly treated with methotrexate (dose range 7.5–20 mg/week).

In total, 25 (69%) patients achieved a CR, 8 (22%) patients achieved a PR and 3 (8%) patients were considered NR (2 patients with JIA associated anterior uveitis and 1 patient with idiopathic intermediate uveitis).

### Adalimumab Trough Levels and antidrug antibodies (ADA) development

**Fig. 1:**
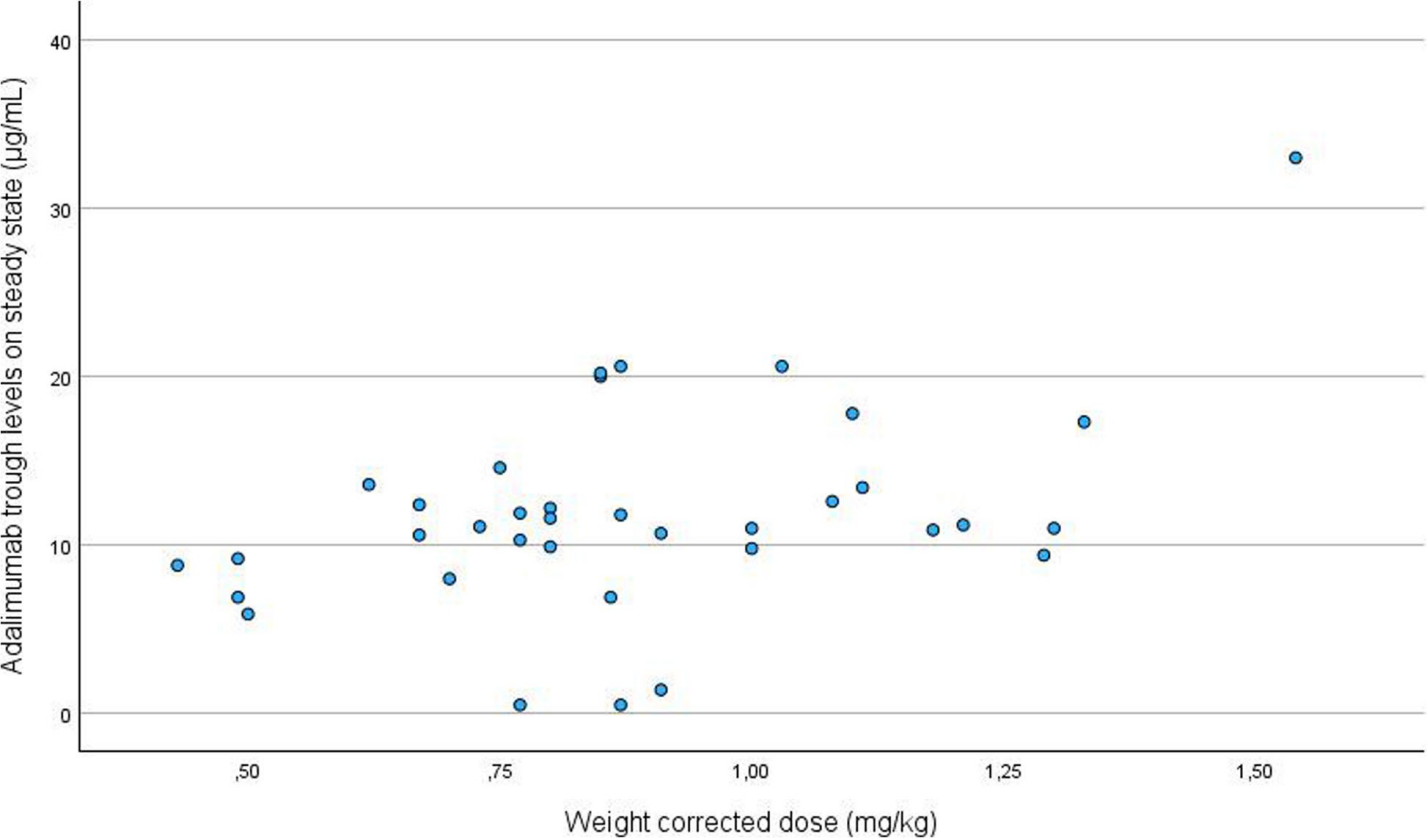
Scatterplot of adalimumab trough levels per adalimumab weight corrected dose

The mean (SD) adalimumab TL was 11.85 µg/mL (range 0 to 33 µg/mL). As shown in Fig. 1, adalimumab TL were highly variable even in patients with comparable weight corrected dosage. ADA were present in 2 (6%) patients (1 patient was a PR, 1 patient a NR).

**Fig. 2:**
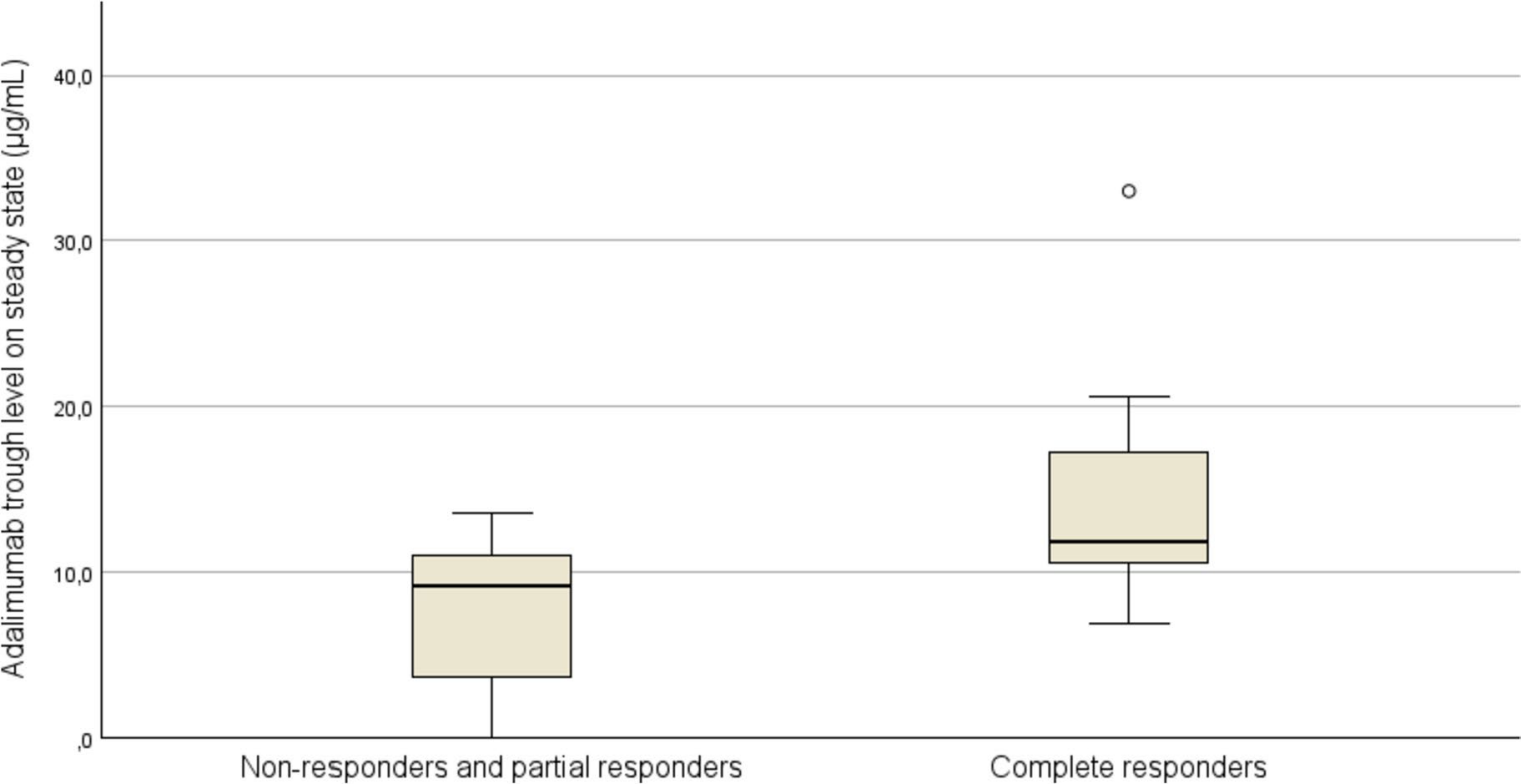
Adalimumab Trough Levels for complete responders, partial responders and non-responders after at least 24 weeks of adalimumab treatment. Boxes represent median values and the first to third quartile. The whiskers extend to the furthest observation within ± 1.5 IQR of the first and third quartile. Observations outside 3 IQR are marked with (°)

When stratified by clinical response, a significant difference in adalimumab TL was observed in CR versus PR and NR (Mann-Withney *P* = 0.004; Fig. 2), with median adalimumab TL of 11.8 (range 6.9–33.0) μg/mL and 9.2 μg/mL (range 0–13.6), respectively. This could not be confirmed when comparing CR to PR (Mann-Withney *p* = 0.03) with median adalimumab TL of 11,8 (range 6.9–33.0) μg/mL versus 9.3 (range 0–13.6) μg/mL, respectively.

Adalimumab TLs did not significantly differ between patients receiving concomitant treatment with MTX and those receiving adalimumab monotherapy: (median 11.8 range (1.4–33) vs. median 10,6 range (0.5–17.8) μg/mL; *p* = 0.089). CR was observed in 66% (14/21) and 73% (11/15) of patients who had and had not received concomitant MTX, respectively (*p* = 0.183). In addition we did not observe any significant influence of sex, age, location of uveitis, uveitis aetiology and SUN score on adalimumab TL (data shown in Additional file 1: Table 1, Figs. 1, 2).

### Defining the therapeutic range for adalimumab trough levels

**Fig. 3:**
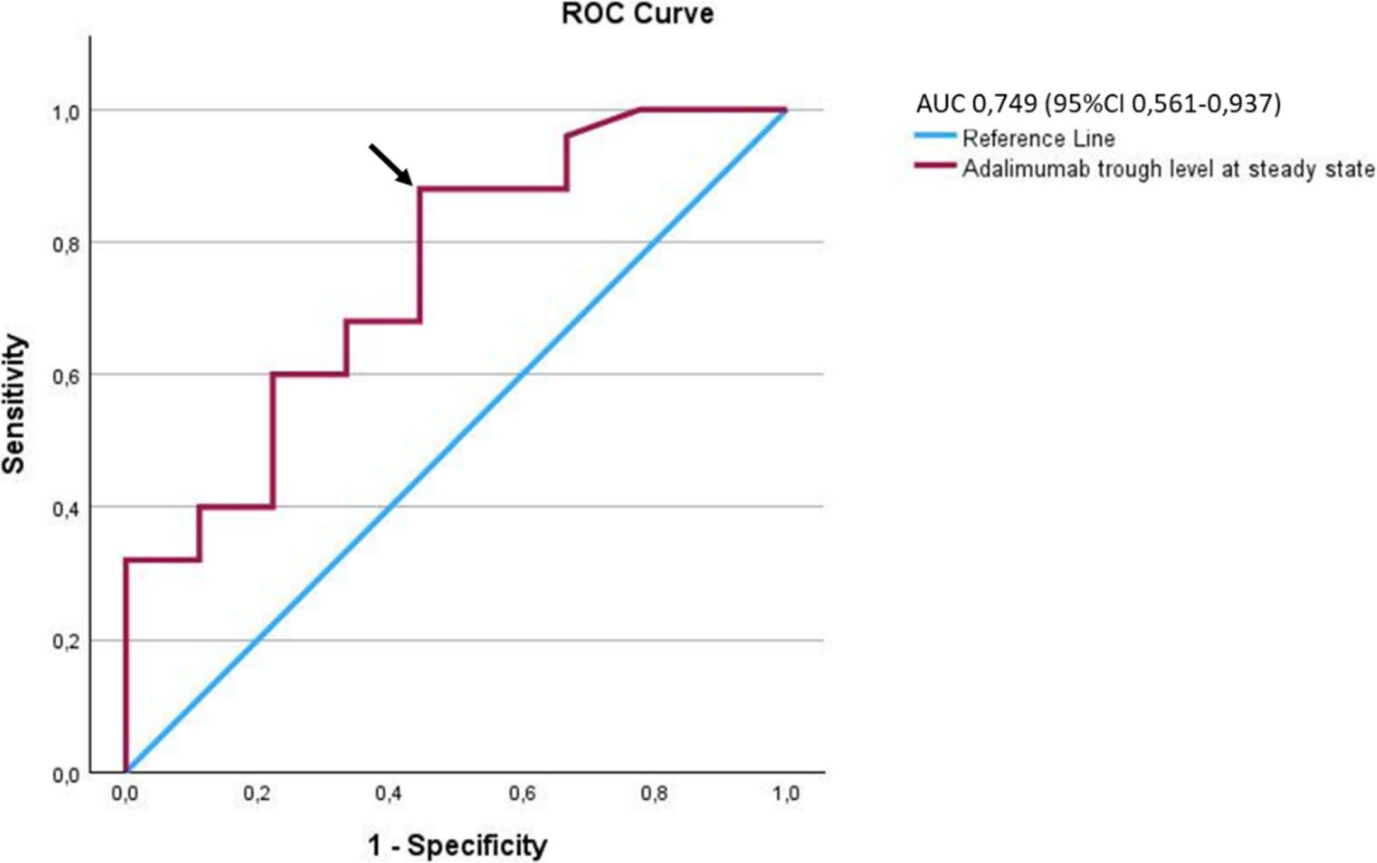
Receiver-Operator Characteristics Analyses. The adalimumab cut-off value corresponding with the most optimal trade-off between sensitivity and specificity is 9.6 µg/mL (arrowhead)

On ROC analysis a minimal effective maintenance adalimumab TL of 9.6 µg/mL was found, with an area under the curve of 0.749 (95% CI, 0.561–0.937), sensitivity 88% and a specificity of 56%; positive predictive value, 85%; negative predictive value, 62.5%) (Fig. 3).

**Fig. 4:**
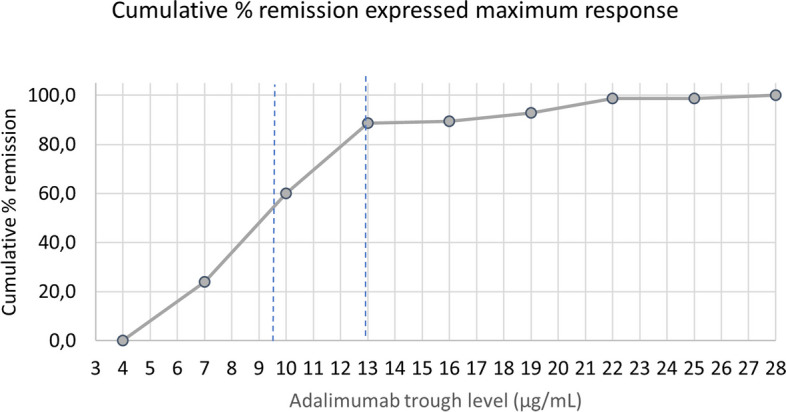
Concentration Effect Curve. Cumulative treatment response by adalimumab concentration through levels. Cumulative % complete remission expressed on the maximum response. Dashed lines indicate the proposed therapeutic interval (9.6 -13 µg/mL)

A concentration-effect curve was created to confirm that clinical response increased with increasing TL. We computed the cumulative % of patients with CR at the specified TL or below (normalized for the maximum of 69.4% CR) and plotted them against the incremental increases (of 3 µg/mL) in adalimumab TL. As shown in Fig. 4, the incremental gain in CR rate reached a near plateau at 13 µg/mL with CR obtained in 88.6% of complete responders. Levels between 13 -16 µg/mL and 16–19 µg/mL only improved clinical efficacy for 0.8% and 3.5% of CR, respectively. Therefore, the upper limit of the therapeutic range was determined at 13 µg/mL.

## Discussion

In this study, which is the first to define a therapeutic range of adalimumab based on adalimumab TL measurement in 36 patients with childhood NIU treated with standard dosing of adalimumab every other week for at least 24 consecutive weeks, we defined a therapeutic range (9.65–13 µg/mL) that corresponds with a good clinical response.

Adalimumab TL were significantly higher in patients who achieved CR than those who did not (*P* = 0.004). This is in accordance with the findings of previously published data in adults [14, 27] and children with NIU [12, 13]. However, previous paediatric studies, mainly focused on the association of adalimumab TL and ADA with the clinical response, included only children with JIA associated chronic anterior uveitis and cut-off values for obtaining good clinical response were not established.

The mean (SD) adalimumab TL was 11.85 µg/mL with a wide interindividual variability (range 0 to 33 µg/mL), even in patients with comparable weight corrected dosage. Pharmacokinetic (PK) studies in adults and children with other immune-mediated diseases (IMD's) have revealed a significant inter-individual variability in the systemic concentrations of biological drugs [28–30]. The therapeutic outcome is closely related to systemic drug levels, which are influenced by several factors including, but not limited to, anthropometric characteristics, immunogenicity, concomitant treatment with immunomodulators, and genetic factors [16, 30]. Children clearly present unique challenges in accurately dosing these medications given the heterogeneity of this population. Different dosing strategies of adalimumab have been used over the past decade in the treatment of JIA and NIU. Adalimumab, dosing was initially 24 mg/m2 body surface area (BSA) eow but changed to dosing schedule based on weight categories [23]. In the United States; patients weighing 10–15 kg, 15–30 kg, and > 30 kg received a fixed dose of 10, 20, and 40 mg eow, outside the US patients weighing < 30 kg, and ≥ 30 kg received a fixed dose of 20 and 40 mg eow respectively [28, 31]. In our study this dosing schedule resulted in a weight-based dosing variation median (IQR) of 0,85 (0.7 to 1.1) mg/kg body weight, with large interindividual variation of adalimumab TL even for weight corrected doses, supporting the need to include TDM to understand the correlation between dosing, systemic drug exposure, and treatment response. Although the use of body weight and BSA are relatively practical in clinical care, it is not clear what parameters of body composition correlate best with the systemic exposure and treatment response to adalimumab in children [32]. In a recent study Verstegen et al. did not find meaningful differences between body weight and BSA in comparison to the alternative dosing parameters based on ideal body weight, fat free mass, and lean body weight [32]. Neutralising ADA are well recognised for both infliximab and adalimumab and significantly affect drug serum concentrations and also inhibit their ability to bind with TNF-α. The clinical relevance of ADA has again been underlined in a recent systematic review by Doeleman et al. [33]. Because of the low number of patients with ADA we could not investigate the association between presence ADA and drug serum concentrations or diseases activity.

There is controversy whether concomitant MTX treatment influences drug levels and ADA development [16]. In our cohort, 21 (58%) patients were concomitantly treated with methotrexate, without a significant impact on adalimumab trough levels (*P* = 0,089) and outcome.

Pharmacogenetic (PG) studies in immune-mediated pathologies have provided evidence of the influence of certain genetic polymorphisms in the response to biological drugs, although the relevance of these findings in NIU is currently unknown. Thus far few studies have been conducted aimed at evaluating the clinical relevance of PK and PG aspects in NIU [16].

The therapeutic drug ranges for anti-TNF drugs in pediatric IMD’s are not yet defined. For JIA patients, adalimumab TL of ≥ 7–8 μg/ml are reported to be therapeutic [22]. Leinonen et al. were unable to suggest any threshold for therapeutic adalimumab TL in 31 children with JIA-related uveitis since adalimumab TL levels did not associate with the activity of the uveitis [13]. In a recently published observational study of Choi et al. in children with inflammatory bowel disease (IBD), adalimumab TL > 8.76 μg/mL predicted mucosal heeling and histologic remission, most cases of therapeutic failure were associated with low serum drug levels [34]. Adalimumab serum levels that are therapeutic when managing JIA-related uveitis are presently unknown. Our proposed therapeutic range for children with NIU is higher than the ones described in adults with rheumatoid arthritis (RA) (5–8 μg/mL) [18], IBD (8–12 μg/mL) [20] and psoriasis (3.51–7.00 μg/mL) [19]. Additionally our adalimumab threshold is much higher than the 3.3 µg/mL value described by Bellur et al., in a study of 42 adult NIU patients but their specificity and area under the curve were lower [35].

However, it is difficult to extrapolate these adult data to children with IMID’s in general and NIU in particular because of PK differences between adults and children linked to body weight and developmental differences in tissue composition, blood flow rates, enzyme and plasma protein concentrations, and glomerular filtration rate [36]. These differences in physiology influence the concentration of drug within the plasma or tissue [37]. Compared to adults, it has been observed that children have faster weight-normalized plasma clearance of monoclonal antibodies [38]. As a result, trough serum drug concentrations of adalimumab and etanercept appear to be lower in young children despite what appears to be adequate dosing [22]. Additionally, because of differences in targeted tissue, NIU should be viewed differently from JIA and IBD in children. Although the pathophysiology and thus TNF disposition of children with JIA and adults with RA might be similar, as evidenced by similar TNF levels in synovial fluid [39, 40] it is unknown whether the TNF disposition in the eye of children with NIU is comparable to the concentrations in the synovial fluid of children with JIA and intestinal tissue in IBD. The eye resides behind particularly strong blood–aqueous barrier and blood-retinal barrier, located at anterior and posterior segments respectively [41], limiting drug penetration from the blood into the eye, thus reducing its bioavailability in the target site of action [42]. Consequently, after systemic administration of adalimumab, the intraocular concentration is lower than the blood concentration and therefore, patients with NIU may require elevated systemic adalimumab TL to increase intraocular bioavailability and reach a therapeutic effect. Additionally, as it has been suggested that a higher dosage of infliximab, another anti-TNF drug used to treat NIU, is needed to obtain adequate drug levels in the eye [43]. This might be suggestive for the need of higher doses of systemic drugs to achieve therapeutic concentrations within the eye. To date, PK data on adalimumab in the eye are not available. In the CR group, adalimumab TL up to 33 µg/mL were observed. In total 9 of 25 CR had a supratherapeutic TL (data shown in Additional file 2), we hypothesize that in these patients, adalimumab dosing interval might be lengthened, leading to adalimumab TL within the therapeutic range without losing clinical efficacy and saving costs. Therefore, these data support the hypothesis that with the current fixed standard dosing regimen, an important percentage of children with NIU are being over treated. Trough levels below the lower margin of the therapeutic range were observed in 10 out of 36 patients. In patients with no or partial response, with TL below the lower range (7/11 patients of our population) we hypothesize that shortening the adalimumab dosing interval, aiming for adalimumab TL within the therapeutic range, could optimise the clinical efficacy. In patients with no or partial response with undetectable TL and development of high levels of ADA (> 125 ng/mL), we would suggest using another anti-TNFα agent (excluding etanercept). In patients with no or partial response despite TL within the proposed therapeutic range, we would suggest a class switch. In patients with complete response and very low TL, for example below half of the lower margin, we hypothesize that disease activity in these patients is low, as a meaningful clinical effect of these TL could not be expected based upon the established therapeutic adalimumab range. In these cases, one could stop adalimumab treatment under further careful clinical monitoring.

This study is limited to its retrospective design, sample size and the heterogeneity of uveitis diagnoses and anatomical subtypes, a limitation often encountered in studies in the field of paediatric uveitis, therefore limiting the generalization of its results. In addition, median TL instead of single TL from patients might better represent the real TL of the individual patients and therefore would have generated more robust data. However, this was not possible in the current retrospective design. Another limitation is the lack of information on adherence. Adherence to self-injectable biologic therapies is well known to be variable, and rarely 100%. In addition to patient choice, interruption of anti-TNFα agents is often recommended at times of intercurrent infection or before elective surgical procedures. Inevitably, trough drug levels may fall, with the potential consequence of some loss of disease control. Nevertheless, this pilot study, providing one of the largest sample sizes, is the first to define a therapeutic range for adalimumab trough levels corresponding with adequate clinical response in the treatment of childhood NIU. The therapeutic algorithm proposed, based on the current data, needs to be confirmed in prospective patient cohorts, ideally using median TL values instead of single TL per patient.

## Conclusions

This study, the first of its kind performed in pediatric NIU, defines a therapeutic range for adalimumab trough levels (9.6–13 µg/mL), that corresponds with good clinical response. Our proposed therapeutic range is significantly higher than the comparable therapeutic ranges available for adalimumab in adults with different types of IMID’s, suggesting that higher adalimumab trough levels are required in children with NIU to achieve complete remission. Defining a therapeutic range is of utmost importance to allow the composition of a therapeutic algorithm for paediatric NIU, in which the dosing schedule can be adjusted according to serum trough levels of adalimumab and ADA. Applying TDM of adalimumab in clinical practice may optimise treat-to-target strategies in paediatric NIU, resulting in higher response rates and less side effects but also lower the treatment-associated costs. As a result, patients might have improved visual outcomes with less long-term disability.

### Supplementary Information

**Additional file 1: Table 1.** Influence of different variables on adalimumab TL. **Figure 1.** Correlation between adalimumab TL and age. **Figure 2.** Correlation between adalimumab TL and SUN score.

**Additional file 2:** Distribution of the clinical response groups in relation to the therapeutic range.

## Data Availability

Datasets used and/or analysed during the current study are available from the corresponding author on reasonable request.

## Abbreviations

ADA: Anti-drug antibodies
CEC: Concentration effect curve
CME: Cystoid macular oedema
CR: Complete responders
IMID's: Immune mediated diseases
JIA: Juvenile idiopathic arthritis
NIU: Noninfectious uveitis
NR: Non-responders
OCT: Optical coherence tomography
PR: Partial responders
SUN: Standardization of uveitis nomenclature
TDM: Therapeutic drug monitoring
TL: Trough levels
