# Supplementary material for: Defining a therapeutic range for adalimumab serum concentrations in the management of pediatric noninfectious uveitis, a step towards personalized treatment
Source: Pediatr Rheumatol Online J. 2023 Dec 20;21:148. doi: 10.1186/s12969-023-00928-2 (PMC10734081; doi:10.1186/s12969-023-00928-2)
Supplement: Supplementary file 1 — Additional file 1: Table 1. Influence of different variables on adalimumab TL. Figure 1. Correlation between adalimumab TL and age. Figure 2. Correlation between adalimumab TL and SUN score. [file 12969_2023_928_MOESM1_ESM.docx]

**Supplementary material file 1**

**Table.1. Influence of different variables on adalimumab TL.**

|  |  | median TL (range) |  |
| --- | --- | --- | --- |
| Sex | Male (n=14)  Female (n=22) | 10.2 (1.4-20.6)  11.9 (0.5-33) | P=0.160 |
| Location of uveitis | Anterior (n=24)  Non-anterior (n=12) | 11.0 (0.5-33)  11.2 (1.4-20) | P=0.779 |
| Etiological diagnosis | JIA-associated (n=19)  Non-JIA associated (=17) | 11.0 (0.5-33)  11.1 (0.5-20) | P=0.490 |

**Figure 1. Correlation between adalimumab TL and age.**

**
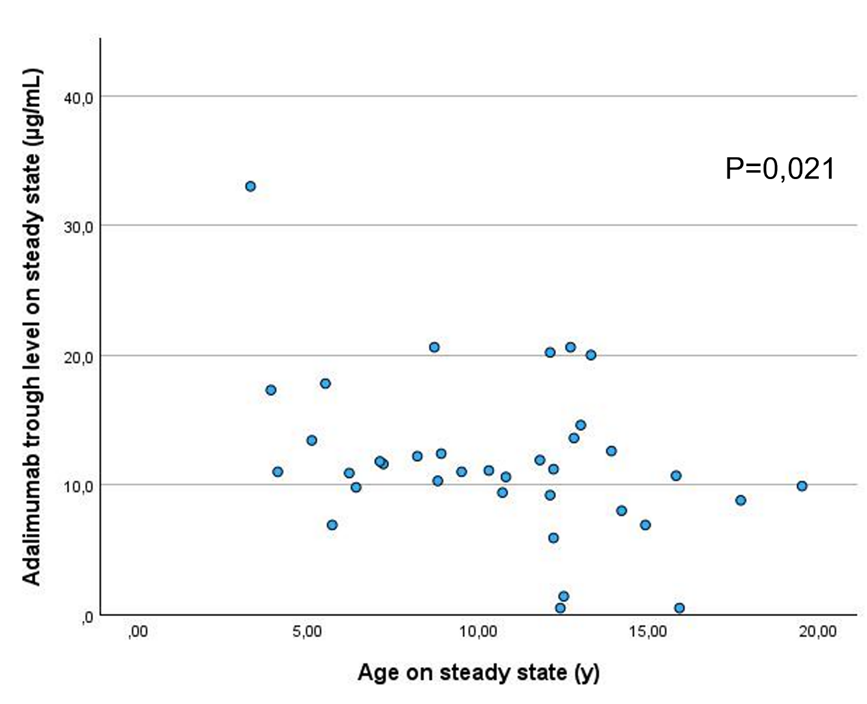
**

**Figure 2. Correlation between adalimumab TL and SUN score.**

**
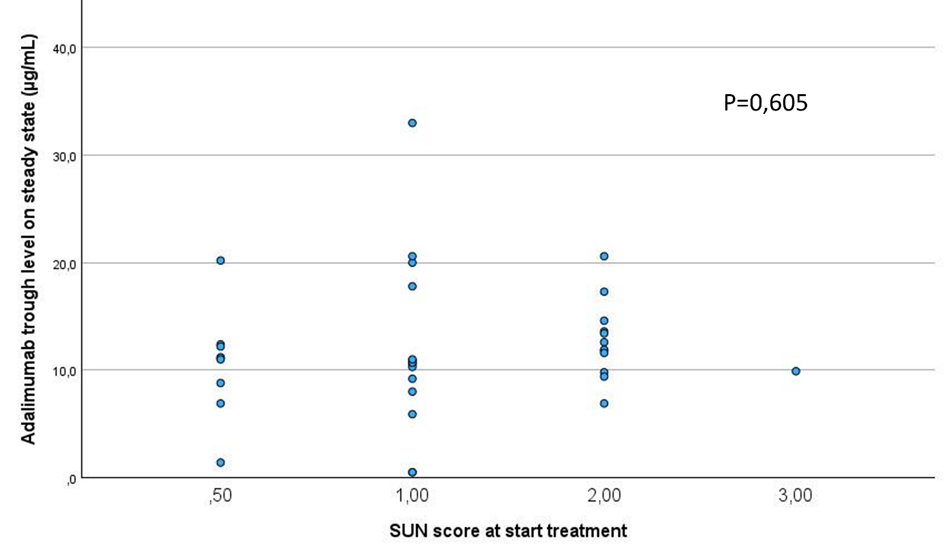
**
