# Supplementary material for: Defining a therapeutic range for adalimumab serum concentrations in the management of pediatric noninfectious uveitis, a step towards personalized treatment
Source: Pediatr Rheumatol Online J. 2023 Dec 20;21:148. doi: 10.1186/s12969-023-00928-2 (PMC10734081; doi:10.1186/s12969-023-00928-2)

**Supplementary material file 2**

**Distribution of the clinical response groups in relation to the therapeutic range.**

CR with supratherapeutic drug levels (indicated in green) are eligible to lengthen adalimumab dosing.

Non-CR with subtherapeutic drug levels (indicated in red) are eligible to shorten adalimumab dosing interval.


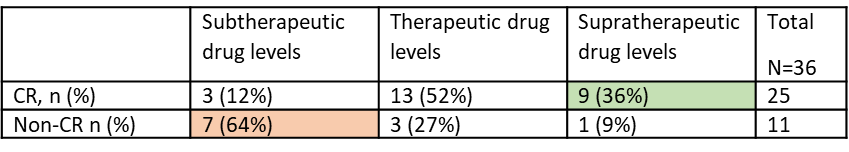

Supplement: Supplementary file 2 — Additional file 2: Distribution of the clinical response groups in relation to the therapeutic range. [file 12969_2023_928_MOESM2_ESM.docx]
